# Genes from scratch – the evolutionary fate of *de novo* genes

**DOI:** 10.1016/j.tig.2015.02.007

**Published:** 2015-04

**Authors:** Christian Schlötterer

**Affiliations:** Institut für Populationsgenetik, Vetmeduni, Veterinärplatz 1, 1210 Wien, Austria

**Keywords:** orphans, *de novo* genes, transcription, population genetics

## Abstract

Although considered an extremely unlikely event, many genes emerge from previously noncoding genomic regions. This review covers the entire life cycle of such *de novo* genes. Two competing hypotheses about the process of *de novo* gene birth are discussed as well as the high death rate of *de novo* genes. Despite the high death rate, some *de novo* genes are retained and remain functional, even in distantly related species, through their integration into gene networks. Further studies combining gene expression with ribosome profiling in multiple populations across different species will be instrumental for an improved understanding of the evolutionary processes operating on *de novo* genes.

## Highlights

• *De novo* genes frequently arise from noncoding DNA.
• While most of the *de novo* genes are lost, a fraction of them becomes essential.
• *De novo* genes are most likely involved in the response to a rapidly changing environment.

## Are orphan genes a dated concept?

For many years, it had been considered extremely unlikely, if not impossible, that genes with no detectable homology could emerge (e.g., [1]). With the availability of the full genomic sequence of yeast, however, this picture changed. About one third of the entire set of genes in baker's yeast has no sequence similarity to genes from other organisms [2]. Because nothing was known about their ancestors, these new genes were termed orphans (or ORFans in the microbial world [3]).

It has become common practice to identify orphan genes based on sequence similarity searches (e.g., BLAST) using a very relaxed significance cutoff: those genes with no hit in other species are classified as orphans [4]. The term orphan was not only appealing but also precise as long as only a few sequenced genomes were available. With an increasing number of sequenced genomes, the taxonomic sampling became denser and the definition of orphans lost its precision: orphans could now be detected in related species, leading to a violation of the definition. To account for this, it has been proposed that orphans be renamed as taxonomically restricted genes [5], but this concept requires an often arbitrary definition of the taxonomic depth to distinguish the relevant units.

## Mechanisms giving rise to orphans

Given this imprecision, it may be more informative to focus on the biological processes generating orphan genes. When the definition of orphan genes is relaxed such that some sequence similarity of orphans with other genes is permitted, processes like exaptation of transposable elements, gene duplication, and horizontal gene transfer emerge as potential forces underlying the generation of orphan genes [6]. Genes originating from such processes with detectable sequence similarities are better characterized as young genes and should be clearly distinguished from orphan genes *sensu stricto*. Mechanisms resulting in true orphans can be placed into four categories, which I outline here. (i) Origin of new genes from previously noncoding DNA – these genes have also been called *de novo* genes indicating that the ancestral sequence was not functional. (ii) Gene duplication and rapid divergence: either gene duplications or insertions of reverse transcribed mRNA sequences into the genome result in duplications of already existing genes. It has been proposed that duplicated copies may undergo phases of rapid evolution in a combination of neutral and adaptive changes [4]. This rapid evolution erases the sequence similarity with the other copies, generating an orphan gene. Despite being conceptually appealing, this class of orphan genes is difficult to distinguish from *de novo* genes because it is very challenging to identify historically rapidly evolving sequences. Hence, I treat this class jointly with *de novo* genes. (iii) Horizontal gene transfer: integration of foreign DNA from bacteria or viruses into the host genome may result in the acquisition of hitherto absent genes. Given the vast number of viral sequences, it is very likely that the source of the acquired gene has not yet been sequenced. Although this mechanism is prevalent in prokaryotes, based on the current surveys of orphan genes in eukaryotes, very little support for horizontal gene transfer has been found [6]. (iv) Frameshift mutations (overprinting): N-terminal frameshifts could generate an entirely different protein with almost no change in the protein coding DNA sequence (CDS) [7]. In viruses, *de novo* genes are frequently generated without frameshifts in the ancestral gene [8]. Although up to 7% of the orphan genes may originate by this process [9], I suggest their evolutionary dynamics be treated separately because their emergence is frequently coupled with the loss of the progenitor gene.

## Shifting the focus from orphans to *de novo* genes

Given the diversity of processes underlying orphan births and the uncertainty surrounding orphan definition, I propose that future studies describing the patterns of molecular evolution focus solely on *de novo* genes. The unambiguous definition of *de novo* genes will be of key importance for informative meta analyses providing a general picture of the evolutionary dynamics of these genes. The importance of separating novel genes according to the underlying molecular mechanism is emphasized by their previously documented different evolutionary dynamics [10].

## Are *de novo* genes real?

*De novo* genes arise from previously noncoding DNA, are short, and are expressed at low levels [10–12]. These features frequently raise doubts about the biological significance of *de novo* genes. In light of these concerns, several approaches have been used to distinguish true *de novo* genes from random noise.

### Neutrality tests

Molecular evolutionary theory provides an excellent theoretical framework for the identification of functionally important sequences [13]. As purifying selection operates against deleterious mutations, functionally important genes have either a low frequency of or even no such mutations, but this is not the case in stretches of neutrally evolving sequences. Protein coding sequences provide a particularly powerful method to detect purifying selection: if the number of putatively deleterious nonsynonymous mutations is significantly smaller than the number of approximately neutral synonymous substitutions, this indicates a functional gene [13]. Although old genes show a more pronounced signal of purifying selection, *de novo* genes differ significantly from noncoding sequences in interspecific [12,14] and intraspecific [12,15] analyses, strongly suggesting that *de novo* genes are subjected to purifying selection. The codon usage of *de novo* genes is another feature that has been attributed to selection. Contrary to neutrally evolving sequences, several studies have demonstrated that preferred codons are enriched in *de novo* genes (Box 1). With selection on codon usage being weak [16,17] and optimization of codon usage being a slow process [18], it appears unlikely that codon usage has been optimized after the *de novo* gene emerged. Rather, it may be that the preferred usage of optimal codons facilitates the emergence of *de novo* genes, specifically their translation.

### Gene expression: RNA and protein

The presence of open reading frames (ORFs) alone is not sufficient evidence for a functional gene. Therefore, many studies use mRNA expression as an indicator for functional *de novo* genes. Given that a large fraction of the genome is transcribed [19], several researchers additionally validated the translation of these mRNAs into proteins by the presence of the corresponding peptides in databases. Such databases are biased towards larger proteins, however, and *de novo* genes are short. This bias has motivated the use of other methods, such as ribosome profiling to study the translation of putative *de novo* genes [14]. Overall, functional importance of *de novo* genes is well-supported by the combined evidence from mRNA and protein expression [14,15,20,21].

### Regulation of gene expression

Another method for assessing whether or not a *de novo* gene is functional rests on the assumption that the modulation of gene expression patterns reflects functional requirements. To this end, several studies have shown that *de novo* genes are not constitutively expressed, but exhibit clear patterns of regulated gene expression (e.g., [9,21–24]). Liu *et al.* [25] not only studied differential regulation of *de novo* genes during the development of *Drosophila melanogaster* embryos, but also identified some developmental stages that were enriched for the expression of *de novo* genes, suggesting that *de novo* genes may preferentially acquire functional roles during some developmental stages.

### Reverse genetics

The most stringent proof of the functional relevance of *de novo* genes comes from reverse genetics. In *Drosophila*, about 30% of young genes are essential, and constitutive silencing them mainly affected the pupal stage [26]. Constitutive knockdown of *de novo* genes also resulted in lethality: out of 11 genes tested, six had an effect on viability [22,26]. Using a tissue-specific knockdown, three out of 33 *D. melanogaster*-specific *de novo* genes induced a bristle-related phenotype [25,27]. Similarly, reverse genetics has also validated the functional importance of *de novo* genes in mice [23]. Taken together, these reverse genetics experiments provide the ultimate proof that *de novo* genes are functional entities rather than a random pattern occurring by chance only.

## Birth of *de novo* genes

**Figure 1:**
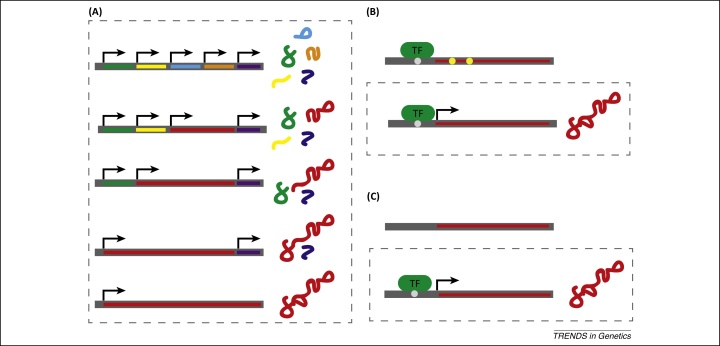
Two competing models of *de novo* gene birth. Open reading frames (ORFs) are shown as colored blocks. Active transcription is symbolized by an arrow and the presence of translation by a peptide. Non-neutral phases are indicated by a broken box. **(A)** and **(B)** illustrate two versions of the expression first model. (A) The protogene model assumes that several short peptides are expressed and during the course of evolution they are combined into a larger *de novo* gene. (B) the ORF contains premature stop codons (yellow circles), which prevent the translation of the expressed mRNA; only after new mutations generate a full-length ORF is the functional *de novo* gene obtained. **(C)** The ORF first model states that a fully functional ORF is present but not expressed because the necessary regulatory signals are missing. Once new mutations generate functional transcription factor (TF) binding sites, the *de novo* gene is expressed and translated.

The emerging picture across different species is that *de novo* genes emerge at high rates [4,6,12]. The birth of *de novo* genes encoding functional proteins involves two important steps: the acquisition of an ORF and the addition of regulatory signals needed for transcription. The sequence of events is not clear, however, and evidence for both models can be found in the literature (Figure 1).

### Expression first

It is now widely accepted that a large fraction of the genome is being transcribed, with many long-noncoding RNA molecules being generated [28,29]. Interestingly, a considerable fraction of these RNAs are also associated with ribosomes [28,30,31], suggesting they are actively translated. Such short peptides form proto genes [14], which can be subject to selection. Through the acquisition of new mutations, proto genes can grow and result in functional *de novo* genes [14] (Figure 1A). Alternatively, it is also possible that the full-length transcript is initially interrupted by stop codons, but new mutations generate the full-length ORF of the *de novo* gene (Figure 1B). The appeal of this model is that it builds on the ubiquity of expressed genomic regions and also circumvents the implausibility problem of *de novo* genes, noted by [1]. This model is strongly supported by a range of studies that found transcription preceded the emergence of an ORF and translation [14,22,32].

### ORF first

This model assumes that ORFs are abundant and only await the acquisition of regulatory elements that control transcription and translation. Indeed, in *Drosophila*, about 60% of 800-bp intergenic sequences harbor ORFs of at least 150 bp [33]. Experimental support for this hypothesis comes from the analysis of a *de novo* gene in mice, which suggested that all the essential functional features of the gene *pldi* were already present, but only the acquisition of the transcription has resulted in a functional gene [23]. Because *pldi* is most likely not a *de novo* protein-coding gene but a noncoding RNA gene, this example may not be representative for protein coding genes. Stronger support comes from an elegant, population genetics-based approach in *Drosophila* [33]. The authors analyzed *de novo* genes that were not expressed in closely related species, but had polymorphic expression within a *D. melanogaster* population. As the statistical power of neutrality tests is low for short genes, the authors were not able to provide a formal proof for a selective spread of the expressed *de novo* genes in the *D. melanogaster* population. Nevertheless, two lines of evidence support this interpretation. (i) More strains expressed the *de novo* genes than expected under neutrality. (ii) Consistent with selectively favored spread of the expressed *de novo* genes, the amount of polymorphism around them was lower in individuals carrying the expressed variant than in those with the non-expressed copy. Importantly, because only the expression of a functional gene could confer a fitness advantage, this pattern suggests that a new mutation resulting in the expression of a pre-existing ORF leads to these *de novo* genes becoming functional.

## Death of *de novo* genes

**Figure 2:**
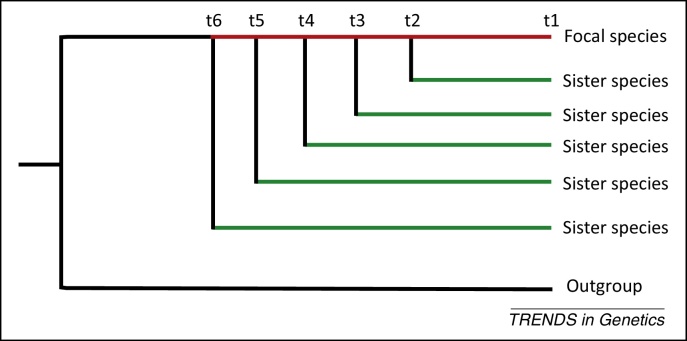
Phylogenetic analysis of *de novo* genes: *de novo* genes are identified in one focal species and their age is determined by the presence of an ortholog in sister taxa (red line). Using the parsimony criterion, the origin of the *de novo* gene is set to the most recent common ancestor of the focal species and the most diverged sister species. The evolutionary stability of *de novo* genes can be studied in those lineages that diverged after the origin of the *de novo* gene (green lines).

The high rate of *de novo* gene birth [4,6,12] in combination with a rather constant number of genes over time [4] predicts that many of the *de novo* genes have only a short lifetime. Testing this prediction, however, requires a phylogenetic framework, which includes a range of species with different evolutionary distances [12,14,22]. Starting from one focal species, the origin of *de novo* genes can be dated by applying the parsimony principle to the presence of the *de novo* genes in the species studied (Figure 2). Once the birth of the *de novo* gene has been dated, its evolutionary dynamics can be studied in species that diverged subsequently (Figure 2). Although lineage-specific mutation patterns and rates are certainly interesting, the ability to study loss-of-function mutations (premature termination codons) and thus the death of *de novo* genes is the greatest benefit of this analysis [12]. Using this approach it has been shown that the probability of loss-of-function mutations is higher for *de novo* genes than for old genes [12,34]. This high death rate of young *de novo* genes explains why the total number of genes remains relatively constant despite the well-documented high rate of *de novo* gene birth [12]. By contrasting conserved *de novo* genes to those that acquired disabling mutations it was found that GC content, gene length, and expression level were positively correlated, and microsatellite number negatively correlated, with sequence conservation [12]. Particularly striking was the observation that *de novo* genes with male-biased gene expression were less likely to acquire premature termination codons. This differential conservation may explain why previous studies identified a high number of *de novo* genes based on gene expression in testis [35,36] or showed an excess of *de novo* genes with male-biased gene expression [37].

## *De novo* genes in action

Several strategies have been pursued to explore the functional contribution of *de novo* genes. Potentially, the most rewarding approach has been the analysis of gene expression. Putative *de novo* genes were found to show a higher gene expression response to abiotic and biotic stressors in *Arabidopsis thaliana* than young genes with a different evolutionary origin [9]. Surprisingly, this signal was restricted to *de novo* genes originating before the *A. thaliana* and *A. lyrata* split [9]. Similarly, putative *de novo* genes in *Daphnia magna* are twice as likely to be differentially expressed under biotic and abiotic stress than old genes [24]. Comparing genes of different ages in yeast, *de novo* genes, and their precursors were enriched for binding of transcription factors related to stress and mating [14]. Finally, *de novo* genes had more pronounced expression differences in a comparison of two *D. melanogaster* populations collected from different environments [33]. This striking similarity across different species strongly suggests that *de novo* genes are particularly important for population-specific responses to biotic and abiotic stresses.

The function of *de novo* goes beyond stress response, however, as they were also shown to serve a vital role during developmental processes [25]. One particularly interesting role was found in *Drosophila*, where many *de novo* genes are related to male reproductive processes [33,35–38]. This class of genes shares an interesting feature with immune-related genes in that they may be involved in an arms race caused by male–male and male–female conflicts [39,40].

## Integrating *de novo* genes into already existing networks

The roles of *de novo* genes that were discussed above are mostly related to functions that require rapid change. Thus, the short persistence times of *de novo* genes nicely fits their functional role. Nevertheless, some *de novo* genes quickly become essential [22,26] and persist for longer time spans. This raises the question of how *de novo* genes could become essential. The prevailing hypothesis is that *de novo* genes become integrated into already existing networks. The first step is the integration into regulatory networks, primarily through acquisition of promoters [41]. The analysis of retroposed genes indicated that regulatory elements can be acquired rapidly from nearby genes or more distant positions in the genome [42]. With increasing age of the regulatory landscape of *de novo* genes, a higher level of complexity is developed through the gradual acquisition of regulatory motifs [41]. The integration of *de novo* genes into protein–protein interaction networks is significantly slower [41]. It has been proposed that protein promiscuity (i.e., non-specific interaction) provides the basis for novel protein–protein interactions [43]. Once established, natural selection will favor beneficial protein–protein interactions and incorporate changes stabilizing them. Interestingly, *de novo* genes were found to interact preferentially, but not exclusively, with genes of the same age [10]. Most likely, *de novo* genes do not acquire catalytic functions, suggesting that they serve primarily regulatory functions in their networks [44].

## Concluding remarks: next steps towards understanding the evolution of *de novo* genes

While past research has proven that genes can originate *de novo* and may even acquire essential functions, the process of *de novo* gene genesis deserves more attention, as does their functional characterization.

Until recently, the evolution of *de novo* genes had been mainly studied in the framework of comparative genomics. However, because the processes of *de novo* gene birth and death occur on the population level, population genetic approaches will be central to understanding these processes. Population genetic theory provides an analytical framework for the interpretation of the selective forces operating on nascent genes. Thus, the combined population genetic analysis of DNA sequences, gene expression, and ribosomal profiling data in multiple individuals will shed light on the selective pressures exerted on each of these levels. Extending this analysis to multiple populations from ecologically distinct habitats as well as additional closely related species holds great promise to determine the evolutionary forces determining the birth and death of *de novo* genes. Experimental evolution in combination with whole genome re-sequencing (evolve and re-sequence, E&R [45]), may provide an opportunity to test the selective advantage of *de novo* genes under controlled laboratory conditions.

## Box 1 Features of de novo genes

• **Short open reading frames (ORFs)** [7,12,46]: the ORFs of *de novo* genes are shorter than those of old genes, but longer than expected by chance [33].
• **Fewer exons** [7,12]: *de novo* genes have fewer exons than old genes.
• **Microsatellites** [11,12,47]: *de novo* genes are enriched for repetitive sequences.
• **Usage of preferred codons** [11,12]: compared to noncoding sequences, *de novo* genes use the preferred codons more often.
• **Low expression level** [9,12,33]: on average, the expression level of *de novo* genes is lower than that of old genes.
• **Higher tissue specificity** [9,11,35]: the expression of *de novo* genes is more tissue-specific than the expression of old genes.
• **Chromosomal location** [12,33,35]: *de novo* genes are enriched on the X-chromosome, while very young ones that still segregate in a population are under-represented on the X-chromosome.
